# Sorafenib-to-regorafenib sequence-induced atherosclerotic cardiovascular disease: a novel case report

**DOI:** 10.3389/fonc.2025.1615472

**Published:** 2025-08-27

**Authors:** Changli Xu, Xianghua Quan, Qie Guo, Donghua Liu, Cheng Lu, Xue Yang, Wen Xu, Fanbo Jin, Haijun Qu, Hongyan Ji

**Affiliations:** ^1^ Department of Cardiovascular Surgery, The Affiliated Hospital of Qingdao University, Qingdao, Shandong, China; ^2^ Department of Pharmacy, The Affiliated Hospital of Qingdao University, Qingdao, Shandong, China

**Keywords:** tyrosine kinase inhibitors, sorafenib, regorafenib, hepatocellular carcinoma, atherosclerotic cardiovascular disease, drug-related toxicity

## Abstract

This case report describes a 59-year-old male with HBV-associated hepatocellular carcinoma who developed progressive atherosclerotic cardiovascular disease (ASCVD) during sequential treatment with sorafenib and regorafenib. Initial sorafenib therapy (400 mg twice daily) led to hand-foot syndrome, necessitating dose reduction, and subsequently resulted in the onset of hypertension (152/92 mmHg), proteinuria (3+), and microscopic hematuria (2+). Due to disease progression, the patient was transitioned to regorafenib (160 mg daily), during which time he experienced worsening vascular toxicity manifested as lower extremity arterial occlusion, non-ST elevation myocardial infarction, and cerebral ischemia. Angiography revealed critical multivessel coronary disease (95-99% left anterior descending artery [LAD] stenosis) and complete femoropopliteal occlusion, requiring revascularization procedures. Notably, these severe ASCVD manifestations occurred despite a low baseline cardiovascular risk (10-year ASCVD risk of 4.5%) and the absence of traditional risk factors, underscoring the cumulative atherogenic effects of sequential vascular endothelial growth factor (VEGF) pathway inhibition. This case highlights the importance of continuous cardiovascular monitoring during tyrosine kinase inhibitor therapy, particularly when transitioning between agents.

## Introduction

Sorafenib and regorafenib are multi-targeted tyrosine kinase inhibitors (TKIs) utilized in the management of hepatocellular carcinoma (HCC). These agents primarily exhibit their antitumor effects by inhibiting vascular endothelial growth factor receptor (VEGFR) and platelet-derived growth factor receptor (PDGFR), which suppresses tumor angiogenesis and proliferation (1, 2). Sorafenib is established as the first-line targeted therapy for HCC, whereas regorafenib is generally considered a second-line treatment option following progression or intolerance to sorafenib (3).

The prevalent adverse effects associated with these agents encompass hypertension, hand-foot skin reaction, overwhelming fatigue, and persistent diarrhea (4–7). Atherosclerotic cardiovascular disease (ASCVD) represents a spectrum of cardiovascular disorders stemming from atherosclerosis, such as coronary artery disease (which may present as either fatal or non-fatal myocardial infarction), cerebrovascular ailments, peripheral arterial dysfunction, and aortic atherosclerosis. To this point, the incidence of sorafenib- and regorafenib-induced atherosclerotic cardiovascular diseases remains low (8, 9). In our clinical practice, we encountered a patient who developed lower extremity arterial occlusion, non-ST segment elevation myocardial infarction (NSTEMI), and cerebral ischemia following treatment with these two agents. The detailed case report is presented below.

## Case presentation

The patient was a 59-year-old male, 176 cm in height and weighing 72 kg. He had no prior history of hypertension, diabetes, hyperlipidemia, smoking, or a family history of cardiovascular disease before starting tyrosine kinase inhibitor therapy. One and a half years prior to admission, the patient began experiencing pain, numbness, and intermittent claudication in the left lower limb without any apparent precipitating factors. Eight months earlier, he developed symptoms of chest tightness, dyspnea, and occasional dizziness. Two months before admission, his lower-extremity symptoms significantly worsened, reducing his maximum walking distance to only 200 meters. Consequently, the patient was admitted to the hospital on November 24, 2024, for further evaluation and management.

The patient has a medical history of chronic hepatitis B infection spanning over 30 years. In June 2018, a solid hepatic mass measuring approximately 3.7 cm in diameter was identified during a routine physical examination. Subsequently, an open surgical resection was performed. Postoperative pathological analysis revealed the following findings: (right posterior lobe of the liver) liver tissue exhibiting partial cellular proliferation with mild nuclear atypia, disruption of the normal hepatic plate architecture, and interstitial infiltration arranged in fine trabecular and nest-like patterns, along with interstitial fibrous tissue hyperplasia. The morphological features were highly suggestive of well-differentiated hepatocellular carcinoma. Immunohistochemical staining demonstrated: CK (+), CK7 (+), Hepatocyte (+), Arginase-1 (+), TTF-1 (–), CK19 (–), CK20 (–), and CDX-2 (–), which collectively support the diagnosis of hepatocellular carcinoma. Post-discharge, the patient continued antiviral therapy with entecavir at a dose of 0.5 mg once daily. In June 2020, HCC recurrence was detected, with the lesion measuring approximately 7 × 5.5 × 5 cm, while HBV-DNA levels remained suppressed (< 100 copies/mL). The patient then underwent a second partial hepatectomy. In May 2021, a follow-up examination was carried out, which included a dynamic contrast-enhanced computed tomography (CT) scan of the upper abdomen. The imaging revealed liver cirrhosis and a space-occupying lesion in the right lobe of the liver measuring 4.4 × 3.0 × 3.9 cm, which was highly suggestive of hepatocellular carcinoma. Subsequently, radiofrequency ablation (RFA) was performed as a therapeutic intervention. During hospitalization, laboratory tests revealed the following: fasting blood glucose 4.60 mmol/L, triglycerides(TG) 0.49 mmol/L, total cholesterol(TC) 4.50 mmol/L, High-Density Lipoprotein Cholesterol (HDL-C)1.41 mmol/L, Low-Density Lipoprotein Cholesterol(LDL-C) 2.62 mmol/L, glycated albumin 12.20%, uric acid 289 μmol/L, creatinine 73 μmol/L, urine protein (−), and blood pressure 124/76 mmHg. No abnormalities were detected in the electrocardiogram (ECG). Upon discharge, sorafenib was prescribed at a dose of 400 mg every 12 hours for further management.

In August 2021, the patient developed hand-foot syndrome, leading to a reduction in sorafenib dosage to 300 mg orally every 12 hours. By October 2021, the patient exhibited drug-induced hypertension with elevated blood pressure (152/92 mmHg), along with proteinuria (3+) and hematuria (2+). Given that these adverse effects were attributed to sorafenib therapy, the treatment regimen was adjusted as follows: (1) initiation of a valsartan-amlodipine combination tablet (one tablet daily) for blood pressure management; and (2) further reduction of sorafenib dosage to 200 mg orally every 12 hours. Subsequent laboratory tests conducted in May 2022 revealed serum creatinine levels at 61 μmol/L, with persistent proteinuria (2+) and hematuria (2+).

**Figure 1:**
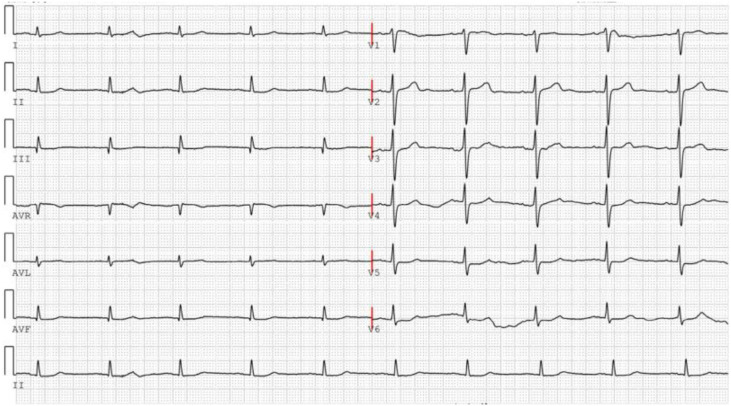
ECG of patient before regorafenib administration.

In May 2023, the patient reported experiencing pain and numbness in the left lower limb. In August 2023, a recurrent tumor measuring 1.4×1.0×1.9 cm was identified, leading to the performance of a repeat RFA procedure. During hospitalization, laboratory test results were as follows: ALT 35 U/L, AST 49 U/L, creatinine 61 μmol/L, uric acid 295 μmol/L, fasting blood glucose 4.62 mmol/L, TG 0.53 mmol/L, TC 4.67 mmol/L, HDL-C 1.32 mmol/L, LDL-C 2.76 mmol/L. The ECG findings are illustrated in **Figure 1**. Due to the insufficient therapeutic response to sorafenib, the treatment regimen was subsequently adjusted to regorafenib at a dose of 160 mg once daily (administered for 5 consecutive days per week with a 2-day rest period during the weekend).

In March 2024, the patient experienced chest tightness, shortness of breath, and dizziness. On October 29, 2024, the numbness and pain in the lower limbs intensified. He was subsequently admitted to the hospital on November 24, 2024. Upon admission, the physical examination revealed the following: urine protein (+), uric acid 285 μmol/L, fasting blood glucose 4.93 mmol/L, TG 0.56 mmol/L,TC 4.27 mmol/L, HDL 1.50 mmol/L, LDL 2.55 mmol/L, and blood pressure 135/77 mmHg.

**Figure 2:**
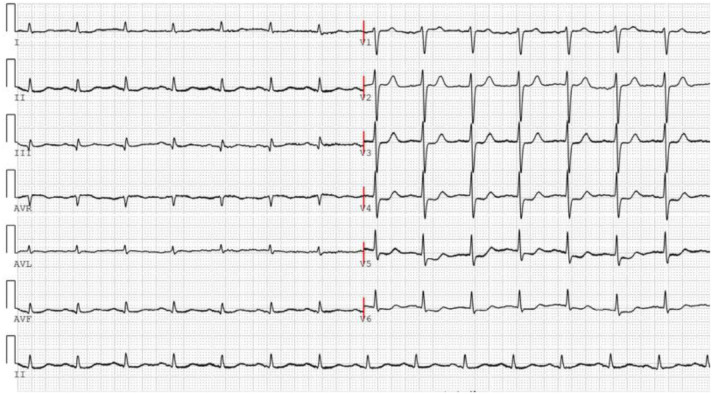
The ECG was performed on November 26, 2024, following the recurrence of the patient’s chest tightness after admission.

**Figure 3:**
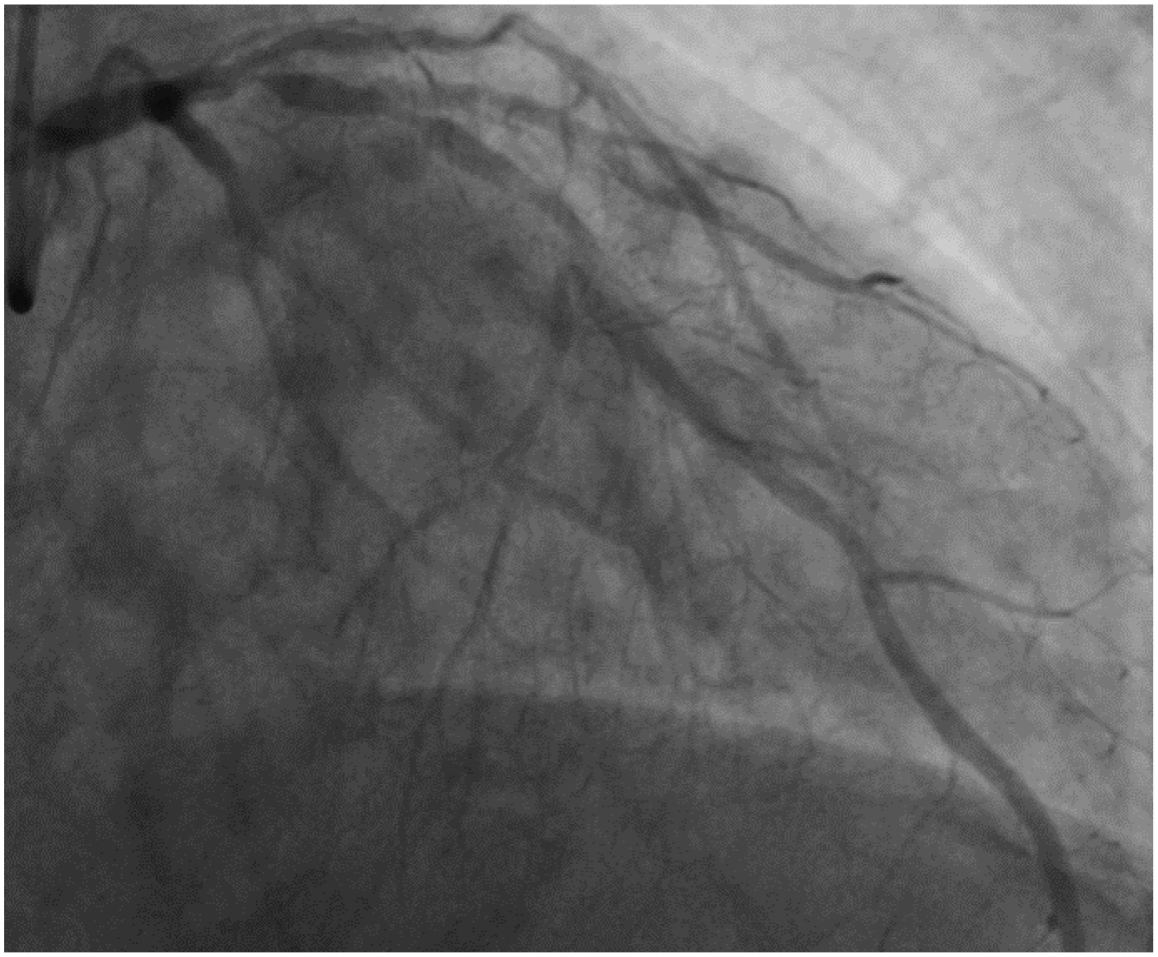
DSA findings of the LAD.

**Figure 4:**
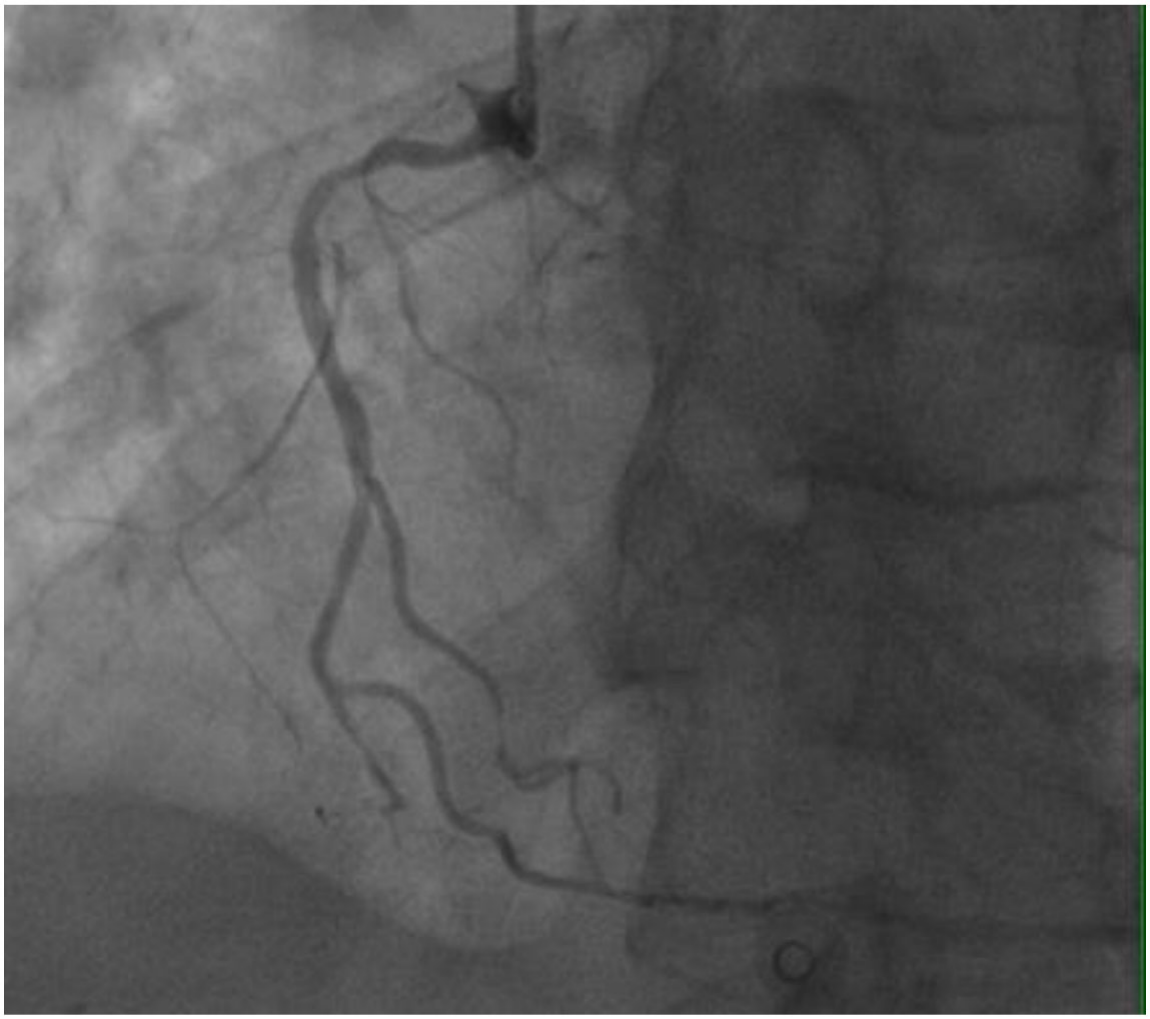
DSA findings of the right coronary artery.

**Figure 5:**
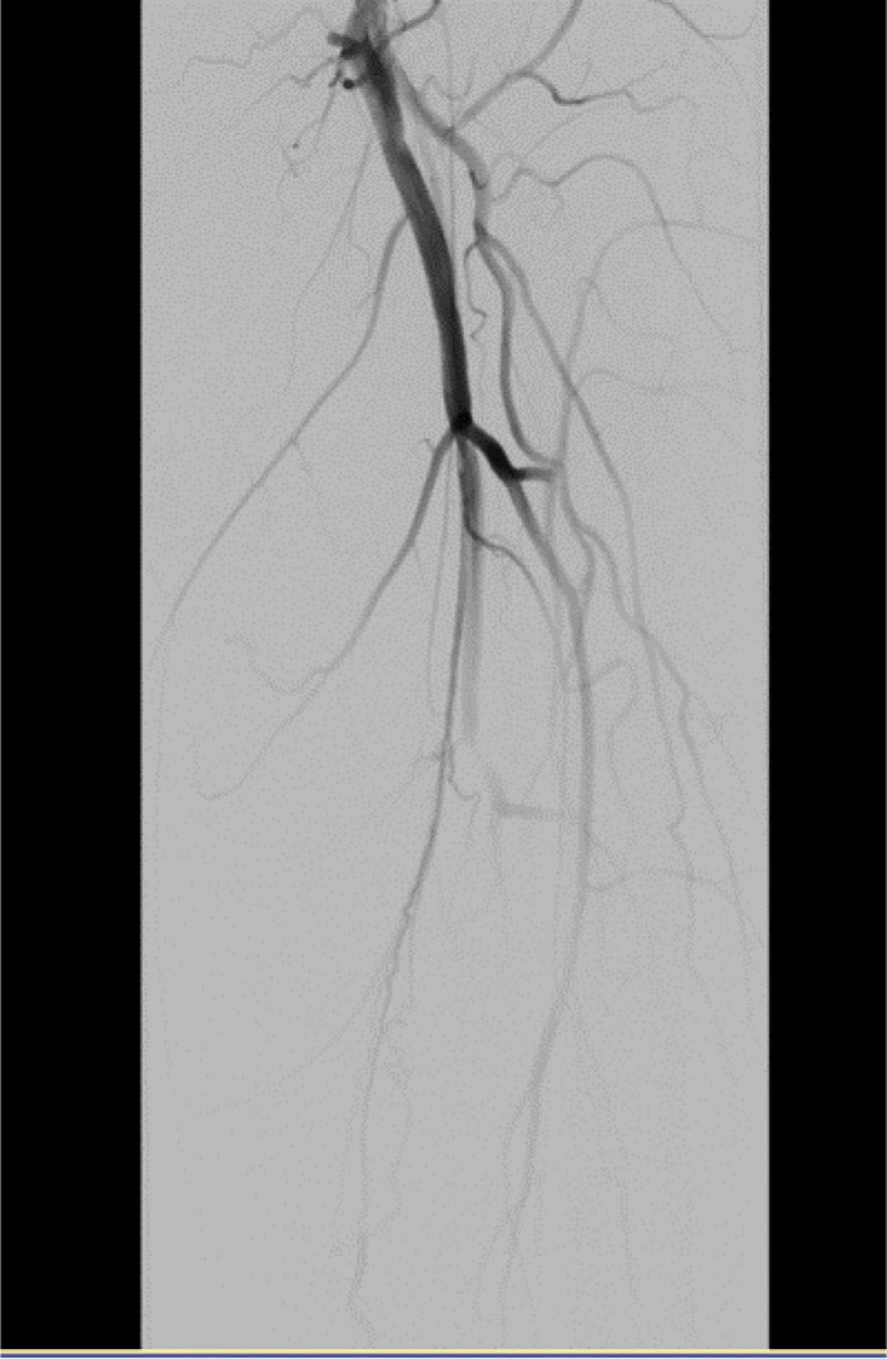
DSA findings of the left femoral artery.

**Figure 6:**
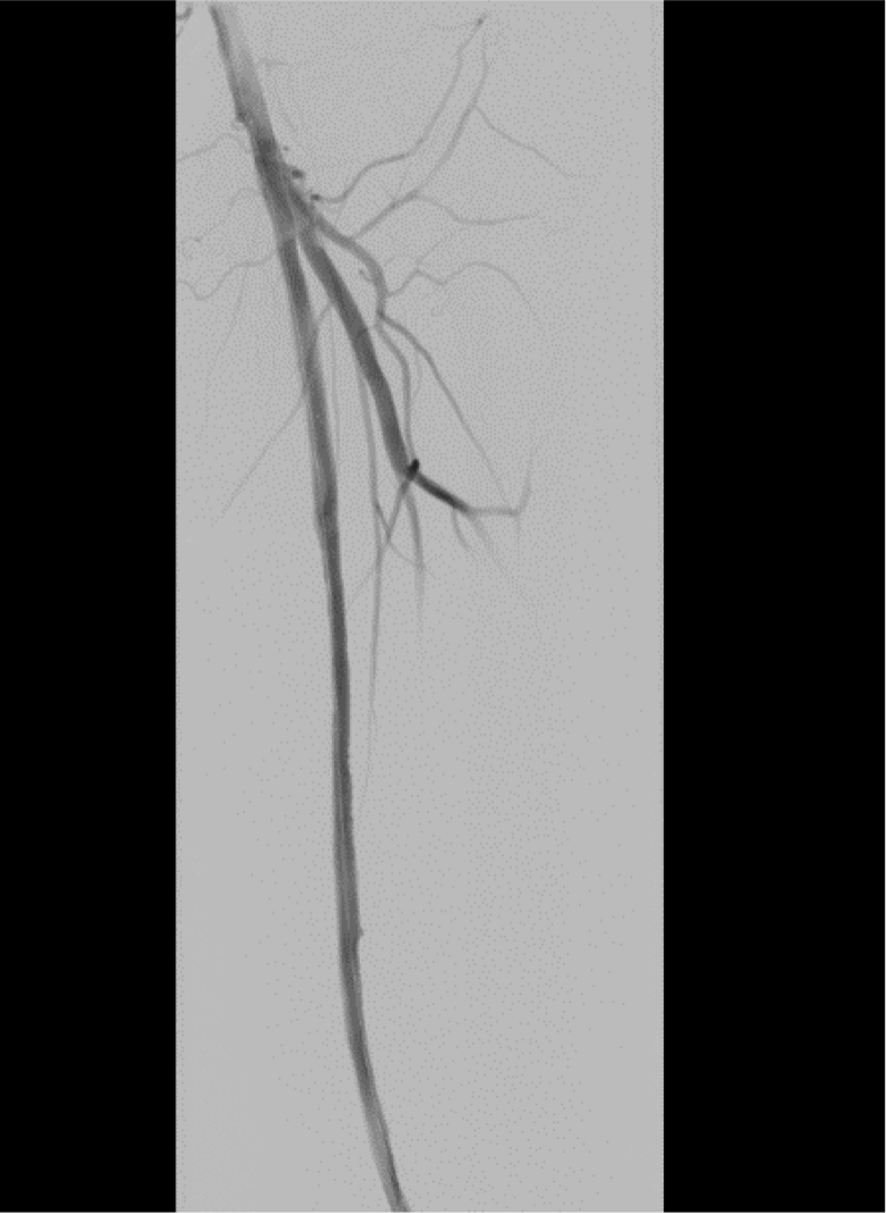
DSA findings following left femoral artery stenting.

**Figure 7:**
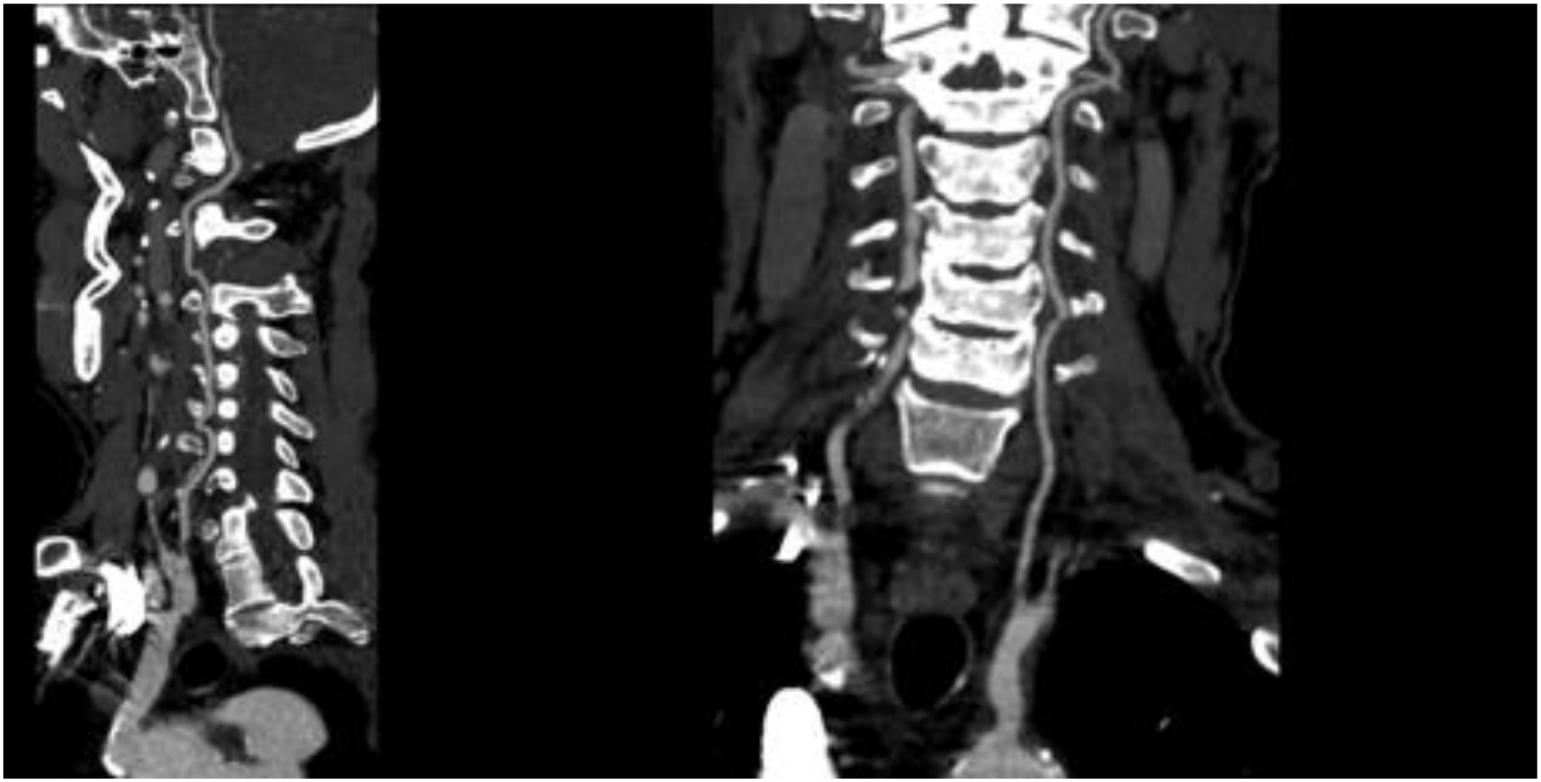
CTA of the cerebral arteries.

**Figure 8:**
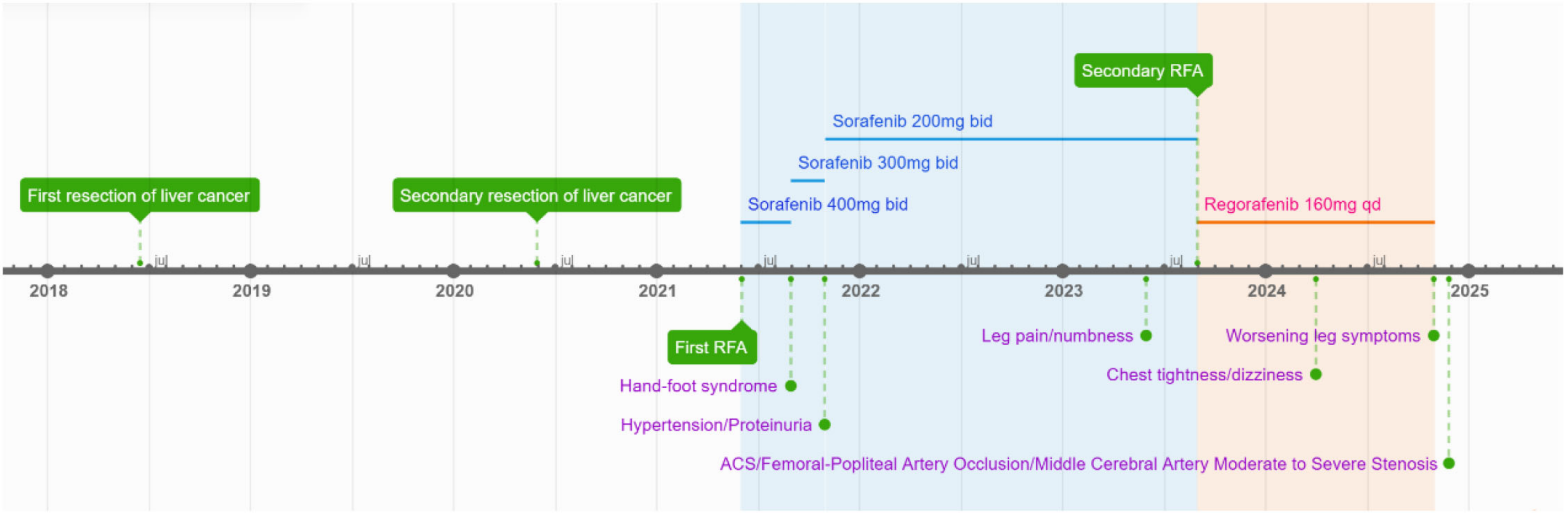
Timeline of clinical events and TKIs therapy.

On November 26, the patient experienced worsening chest tightness and shortness of breath. The EGC (**Figure 2**) revealed abnormal findings, including multi-lead ST-segment depression. Troponin levels were elevated at 0.08 ng/mL (normal range: 0-0.056 ng/mL).Coronary angiography (DSA) demonstrated severe stenosis (95-99%) in the ostium and proximal segment of the left anterior descending artery (LAD) (**Figure 3**), moderate stenosis (30%) in the mid and distal segments of the LAD, significant stenosis (70-80%) in the proximal and mid-segments of the circumflex branch, and moderate stenosis (60-70%) in the proximal and mid-segments of the right coronary artery (**Figure 4**). Additionally, the patient presented with lower extremity atherosclerosis characterized by multiple stenotic lesions and complete occlusion of the left femoral-popliteal artery (**Figure 5**). This condition was treated through the implantation of a lower extremity arterial stent (**Figure 6**). Given the presence of multivessel coronary artery disease, coronary artery bypass grafting (CABG) was performed by the cardiac surgery team. Preoperative cerebral angiography (CTA) showed mild to moderate stenosis of the bilateral internal carotid arteries and wall thickening with moderate to severe stenosis in the M1 segment of the middle cerebral artery (**Figure 7**). Due to the potential association between ASCVD and regorafenib use, regorafenib was discontinued. Following CABG, the patient recovered well and was subsequently discharged from the hospital. The timeline of clinical events and tyrosine kinase inhibitor treatment is presented in **Figure 8**.

## Discussion

Prior to sorafenib treatment, the patient had no traditional risk factors such as hypertension, diabetes, smoking history, or family history of cardiovascular disease. Assessment using the ChinaPAR model indicated that the patient’s 10-year risk of ASCVD was only 4.5%, categorizing him as low-risk for ASCVD development (10). It is important to note that the patient had been regularly taking entecavir for three years before starting sorafenib. Common adverse reactions of entecavir include nausea, fatigue, dizziness, and abdominal pain, with rare cases of renal dysfunction. During these three years of medication, no electrocardiogram abnormalities or myocardial ischemia manifestations were observed. Therefore, the influence of entecavir on the cardiovascular system can be ruled out.

The patient has a 30-year history of chronic HBV infection. However, current evidence-based medical research indicates no significant association between HBV infection and the risk of coronary heart disease (CHD). A 2018 meta-analysis conducted by Wang et al. (11), which included 9 observational studies involving 65,058 HBV-infected individuals and 534,998 controls, revealed that the relative risk (RR) of CHD in HBV-infected patients was 0.99 (95% CI: 0.76-1.22). Consistent findings across all subgroup analyses further support this conclusion.

During sorafenib treatment, the patient sequentially developed common adverse reactions, including hand-foot syndrome, hypertension, and proteinuria. These findings were consistent with the established toxicity profile of multi-target TKIs as reported in the literature (12). Following dose reduction of sorafenib and initiation of valsartan-amlodipine combination therapy for symptomatic management, some symptoms were alleviated. However, after 21 months of treatment, the patient exhibited cardiovascular ischemic manifestations, such as lower limb pain, numbness, and ST-segment changes on electrocardiogram. Given the suboptimal therapeutic response to sorafenib, the treatment was transitioned to regorafenib, which led to a partial reduction in proteinuria. Despite this adjustment, the clinical manifestations of ischemia in the heart, brain, and lower extremities progressively worsened.

Both sorafenib and regorafenib are multi-targeted TKIs that have been extensively documented for their vascular toxicities in numerous studies. The literature demonstrates that sorafenib, by inhibiting targets such as VEGFR and PDGFR, disrupts endothelial cell function and vascular homeostasis, potentially leading to coronary artery spasm or atherosclerosis, which may subsequently trigger acute coronary syndromes (e.g., ST-segment elevation myocardial infarction) (13–15). Similarly, regorafenib, as a broad-spectrum kinase inhibitor, induces cardiovascular toxicity through mechanisms including endothelial dysfunction, microvascular injury, and an increased thrombotic risk (16, 17). Furthermore, TKIs may exacerbate cardiovascular toxicity via off-target effects, including mitochondrial damage, oxidative stress, and endoplasmic reticulum stress (14, 15). Rare cases of ST-segment elevation myocardial infarction associated with sorafenib and regorafenib use have also been reported in the literature (18, 19). Although hepatocellular carcinoma (HCC) is not a direct cause of atherosclerosis, it can contribute to thrombosis and the establishment of a pro-inflammatory microenvironment, characterized by elevated levels of C-reactive protein (CRP), fibrinogen, and interleukin-6 (IL-6). These inflammatory conditions may interact synergistically with tyrosine kinase inhibitors (TKIs)-induced endothelial dysfunction through multiple pathways, thereby accelerating the progression of atherosclerotic cardiovascular disease (ASCVD). This interplay highlights a common pathophysiological mechanism shared between cancer and cardiovascular disease, wherein chronic inflammation serves as a central mediator that simultaneously promotes tumor growth and the development of atherosclerotic plaques (20–22). Given this complex interplay, aggressive management of conventional cardiovascular risk factors (CVRFs)—such as hypertension, dyslipidemia, diabetes, and smoking—is crucial in patients receiving TKIs. Proper control of these modifiable risk factors may help mitigate the compounded cardiovascular toxicity induced by both the malignancy and its treatment, ultimately improving patient outcomes. Notably, the patient exhibited symptoms of lower extremity vascular lesions and mild signs of myocardial ischemia following approximately 24 months of sorafenib therapy, which aligns with the reported delayed onset of atherosclerotic cardiovascular disease (ASCVD) following the initiation of TKIs treatment (8). Although these clinical manifestations are likely attributable to sorafenib-induced vascular injury, their progression during regorafenib therapy suggests either a cumulative toxic effect from continuous VEGF pathway inhibition or a worsening of endothelial dysfunction. This observed clinical pattern highlights the necessity for sustained cardiovascular monitoring even after transitioning to alternative tyrosine kinase inhibitors (TKIs) therapy.

While current guidelines appropriately emphasize intensified monitoring for patients with preexisting cardiovascular risk factors, our case highlights two clinically significant observations: (1) TKI may induce progressive ASCVD independent of baseline risk status through endothelial dysfunction; and (2) this toxicity follows a duration-dependent pattern, typically manifesting after ≥12 months of therapy. Therefore, based on the available evidence (9, 23), we recommend comprehensive cardiovascular monitoring for all patients undergoing treatment with TKI inhibitors, including: (1) baseline assessment (blood pressure, ECG, brain natriuretic peptide (BNP), lipid profile and echo-Doppler of the supra-aortic trunk (SAT)); (2) During active treatment, implement weekly BP monitoring (more frequent if uncontrolled), while performing ECG every 2–4 weeks initially then every 3 months (monthly if QTc >450 ms), echo-Doppler of the SAT should be conducted every 6 months. Importantly, at each follow-up visit, physicians should actively inquire about potential cardiovascular symptoms, with prompt referral to cardiologists if any symptoms emerge.

## Conclusions

Tyrosine kinase inhibitors (TKIs), such as sorafenib and regorafenib, may induce ischemic cardiovascular events through mechanisms involving multi-target inhibition and off-target effects. Although current clinical guidelines have established preventive protocols involving the combination of ACE inhibitors (ACEI)/angiotensin receptor blockers (ARBs) and beta-blockers for traditional chemotherapeutic agents such as anthracyclines, clear regulatory guidance for the management of TKI-associated cardiovascular toxicity remains lacking. Given the distinct vascular toxicity profile of TKIs, the following comprehensive management strategy is proposed: Prior to treatment initiation, a thorough baseline cardiovascular risk assessment should be performed; during therapy, continuous monitoring of cardiovascular symptoms is essential for early detection of toxicity; and upon identification of related symptoms, evidence-based interventions should be promptly implemented (20, 24, 25): 1) ACEI/ARB as first-line therapy for hypertension control (if blood pressure remains uncontrolled despite dose escalation of antihypertensive therapy, dose reduction or temporary discontinuation of TKI may be considered); 2) beta-blockers for tachycardia; 3) statins for dyslipidemia; 4) immediate cessation of TKI therapy in the event of arterial thromboembolic events (ATEs). Concurrently, lifestyle modifications, including regular aerobic exercise, should be integrated into the management plan. This integrated “assessment–monitoring–intervention” model not only accounts for the unique vascular toxicity characteristics of TKIs but also ensures optimal antitumor efficacy and cardiovascular safety, thereby offering a practical framework for clinical decision-making.

## Data Availability

The original contributions presented in the study are included in the article/supplementary material. Further inquiries can be directed to the corresponding authors.
